# Effect of C/N ratio and microelements on nutrient dynamics and cell morphology in submerged fermentation of *Aspergillus giganteus* MTCC 8408 using Taguchi DOE

**DOI:** 10.1007/s13205-017-0611-2

**Published:** 2017-04-13

**Authors:** Debashis Dutta, Mira Debnath Das

**Affiliations:** School of Biochemical Engineering, Indian Institute of Technology (Banaras Hindu University), Varanasi, 221005 India

**Keywords:** *Aspergillus giganteus* MTCC: 8408, Taguchi (DOE) L_8_ orthogonal array (OA), Scanning electron microscopy (SEM), Antifungal protein (afp)

## Abstract

This paper is concerned with the effect of individual Na^+^, K^+^, Mg^2+^ and Ca^2+^ ions upon the nutrient dynamics and cell morphology during submerged fermentation of *Aspergillus giganteus* MTCC 8408 in a medium containing ample soluble starch, corn steep liquor and proteose peptone supply. Nutrient dynamics was elucidated using Taguchi’s DOE (design of experiment) L_8_ orthogonal array (OA) with carbon, nitrogen and four most influensive microelements at their assigned ratio namely; K^+^/Ca^2+^ and Mg^2+^/Na^+^. Scanning electron microscopy (SEM) was used to correlate the effect of selected factors on morphological changes and nutrient dynamics during submerged fermentation. At higher K^+^/Ca^2+^ ratio 4.78:1; the maximum growth was figured out in terms of dry weight, while the inhibition of dry weight production was observed at higher Mg^2+^/Na^+^ ratio 0.94:1 with enhanced intracellular afp production. At lower C/N ratio 27.4:1; filamentous form of growth was maintained with the hyphae being scarcely branched without bulbous cells. Membrane perturbation due to the induction of intracellular oxidative stress was noticed at higher C/N ration. Taguchi DOE resulted in afp yield per substrate utilized, *Y*_p/s_ of 1.08 mg g^−1^ soluble starch utilized with gram dry cell weight (gdcw) of 23.9 g L^−1^ while afp yield per biomass utilized resulted, *Y*_p/x_ of 1.12 mg afp gdcw^−1^ L^−1^ and biomass yield per substrate utilized, *Y*_x/s_ of 1.195 gdcw g^−1^ L^−1^. The present study revealed an opportunity to develop a cost effective methods for cell propagation so as to yield effective inoculum levels towards mass production and formulation studies.

## Introduction

Submerged cultivation of various filamentous ascomycetes is ubiquitous, widespread and used to produce broad-spectrum antimicrobial metabolite, commercially (Papagianni 2004). Nutrient source is a cognitive factor of growth and germination of filamentous fungi (Shah and Tariq 2005). Nutrients exploited in biosynthesis and ATP dependent energy release, assists as fundamental drift towards the viability, survival and sustenance of any organism (Safavi et al. 2007). For commercial cultivation of microorganism, it is imperative to have an in-depth knowledge of their nutritional requirements. The macroelements such as carbon, oxygen, hydrogen, nitrogen, potassium, magnesium, calcium and phosphorus are integral components of carbohydrates, lipids, proteins and nucleic acids and these metabolically active groups are directly/indirectly involved in host-pathogen interaction and self-defense and perpetuation mechanisms. Carbon is required as the skeletal element of all organic molecules, and molecules serving as carbon sources normally also contribute both oxygen and hydrogen. Gao et al. (2007) studied the effect of different C/N ratio on the growth and sporulation of several biocontrol agents. Mg^2+^ ion is widely perceived as an essential element for fungal growth and its biochemical functions (Jasper and Silver 1977) involved in a major way through transducing and the control of cell division in many filamentous ascomycetes through its control of microtubule assembly (Walker 1978). It has also been reported that passage of the Mg^2+^ and Ca^2+^ ions through the plasma membrane shows many of the characteristics of an active transport system with K^+^ ion which extruded to preserve electrical neutrality. The CN ratio is said to significantly affect the number of conidia produced and conidial characteristics as well as on growth, sporulation and biocontrol efficacy (Jackson and Bothast 1990; Jackson and Schisler 1992; Jackson and Slininger 1993; Engelkes et al. 1997). This study on variable nutrient source had been conducted so as to evaluate the fungi for their conidial germination, mycelial growth and sporulating potential on differential media containing various carbon and nitrogen sources and ratios. The culture media which supports better germination growth and sporulation promises to the key to the effective, low cost development of antifungal protein in submerged fermentation. Simple and less expensive media are, therefore, needed to permit their mass-production and commercialization (Liu and Chen 2002; Shah and Tariq 2005). In the conventional approach, an optimization process usually involves one factor at a time (OFAT) (Nandal et al. 2013; Mehta et al. 2016). Conventional optimization procedures such as RSM (response surface methodology) procedures are time consuming, cumbersome, require more experimental data sets and cannot provide information about the mutual interactions of the parameters (Aggarwal et al. 2008). Taguchi design of experiments (DOE) developed by Genichi Taguchi to improve the quality of manufactured goods (Taguchi 1986) has been appreciated in many biotechnology and bioprocess engineering (Ross 1996; Roy 2001) possesses many advantages, includes efficient and logical plan for performing experiments under the consideration of the interactive effects among the control factors.

In this study, we examined and discussed the effects of six most decisive factors viz; soluble starch, corn steep liquor with proteose peptone, K_2_HPO_4_, CaCl_2_, MgSO_4_ and NaCl at their assigned ratio on nutrient dynamics, cell morphology and afp production in submerged fermentation of *Aspergillus giganteus* MTCC 8408. Taguchi DOE was employed with L_8_ OA for enhanced afp production. The aim of this work was also to establish minimum levels for Mg^2+^ in growth medium and to determine whether Ca^2+^ and Na^+^ could act as a growth inhibitor through competition with Mg^2+^.

## Materials and methods

### Strain and culture condition

*Aspergillus giganteus* (CBS-KNAW Fungal Biodiversity Centre, Netherlands; Type: F; Strain Designation No: 515.65), MTCC (Microbial Type Culture Collection) 8408 strain was grown in Czapek’s yeast extract agar slant containing (g L^−1^): K_2_HPO_4_ 1.0; yeast extract: 5.0; sucrose: 30.0; agar: 15.0; NaNO_3_: 0.3; KCl: 0.05; MgSO4·7H_2_O: 0.05; FeSO4·7H_2_O: 0.001; ZnSO4·7H_2_O: 0.001; CuSO4·5H_2_O: 0.0005 for 7 days at 25 °C at initial pH 6.0 and stored at 4 °C for maximum period of 14 days. Unless otherwise stated, all chemicals used in this work were purchased from Merck and Sigma.

### Inoculum preparation and submerged fermentation

In next phase, *Aspergillus giganteus* culture was grown in 60/250 mL Erlenmeyer flask containing Czapek’s yeast extract broth incubated at temperature 25 °C, orbital rotator speed 150 rpm for 24 h at initial pH 6.0, prior to use as inoculum (*A*_600 nm_ ≥ 0.9) for next phase submerged fermentation. Final phase submerged fermentation was carried out in Czapek’s yeast extract broth at their assigned ratio (Table 1) of soluble starch (40, 60 g L^−1^), corn steep liquor, 2% with proteose peptone, 1% (18, 36 g L^−1^), K^+^ ions (1.7214, 5.1724 m-equiv.), Ca^2+^ ions (1.08, 3.2432 m-equiv.), Mg^2+^ ions (3.252, 4.878 m-equiv.), Na^+^ ions (5.1724, 10.3448 m-equiv.) as per Taguchi’s L_8_ OA (Table 2). All experiments were performed in triplicate (mean ± standard deviation of double determination).

**Table 1:** Selection of factors at their assigned level for Taguchi DOE under L_8_ OA

Columns	Factors	Level 1	Level 2
1	C/N ratio	27.4:1	54.9:1
2	K^+^/Ca^2+^	1.59:1	4.78:1
3	INTER COLS 1 × 2	–	–
4	Mg^2+^/Na^+^	0.62:1	0.94:1
5	INTER COLS 1 × 4	–	–
6	INTER COLS 2 × 4	–	–

**Table 2:** Taguchi DOE L_8_ OA with selected factors for Y_p/x_ in submerged fermentation of *A. giganteus* MTCC 8408

Run no.	Columns								
	1	2	3	4	5	6	*Y*_p/x_ (mg gdcw^−1^ L^−1^) ± SD^a^	S/N ratio	Mycelial biomass (gdcw L^−1^ ± SD)^a^
1	1	1	1	1	1	1	0.517 ± 0.0280	−5.769	25.05 ± 0.35
2	1	1	1	2	2	2	0.729 ± 0.0220	−2.758	21.90 ± 0.48
3	1	2	2	1	1	2	0.9045 ± 0.002	−0.872	22.10 ± 0.78
4	1	2	2	2	2	1	0.415 ± 0.0190	−7.667	27.65 ± 0.65
5	2	1	2	1	2	1	0.4015 ± 0.009	−7.934	29.85 ± 0.80
6	2	1	2	2	1	2	0.519 ± 0.0340	−5.753	24.95 ± 0.54
7	2	2	1	1	2	2	0.83 ± 0.01250	−1.621	21.05 ± 0.42
8	2	2	1	2	1	1	0.611 ± 0.0040	−4.280	26.15 ± 0.23

All experiments were performed in triplicate (mean ± standard deviation of double determination)

^a^Mean ± SD (standard deviation) for double determination, gdcw L^−1^ = gram dry cell weight per liter of broth

### Taguchi methodology, data analysis and validation

A statistical tool, Taguchi design of experiments (DOE) was employed to carry out 8 well defined experiments, L_8_ OA with six most influensive parameters (viz. soluble starch, corn steep liquor with proteose peptone, K^+^ ions, Ca^2+^ ions, Mg^2+^ ions, and Na^+^ ions). In this experiment, two level interaction columns (INTER COLS) were designed with outer array to get a total of six interaction. Data obtained were analyzed using Qualitek-4 (Nutek Inc., MI, USA). Taguchi approach could be an efficient and time-saving strategy for parameter optimization in bioprocess development (Dutta and Debntah Das 2016). In this study, the performance quality “larger the better’ was used to define the optimum conditions and evaluated using Eq. (1):

1 $${\text{SN}}_{i} = - 10 \log \left[ {\frac{1}{{N_{i} }}\mathop \sum \limits_{u = 1}^{{N_{i} }} \frac{1}{{y_{u}^{2} }}} \right],$$

where *N*_*i*_, the number of trials for experiment *i* designed at their assigned level, *i*, the experiment number, *u*, the trial number and *y*, experimental value of each trial.

Analysis of the experimental data using the analysis of variance (ANOVA), severity index (SI) and main effects gives the output that is statistically significant in finding the optimum levels.

## Results

### Fermentation factors and their main effect

**Fig. 1:**
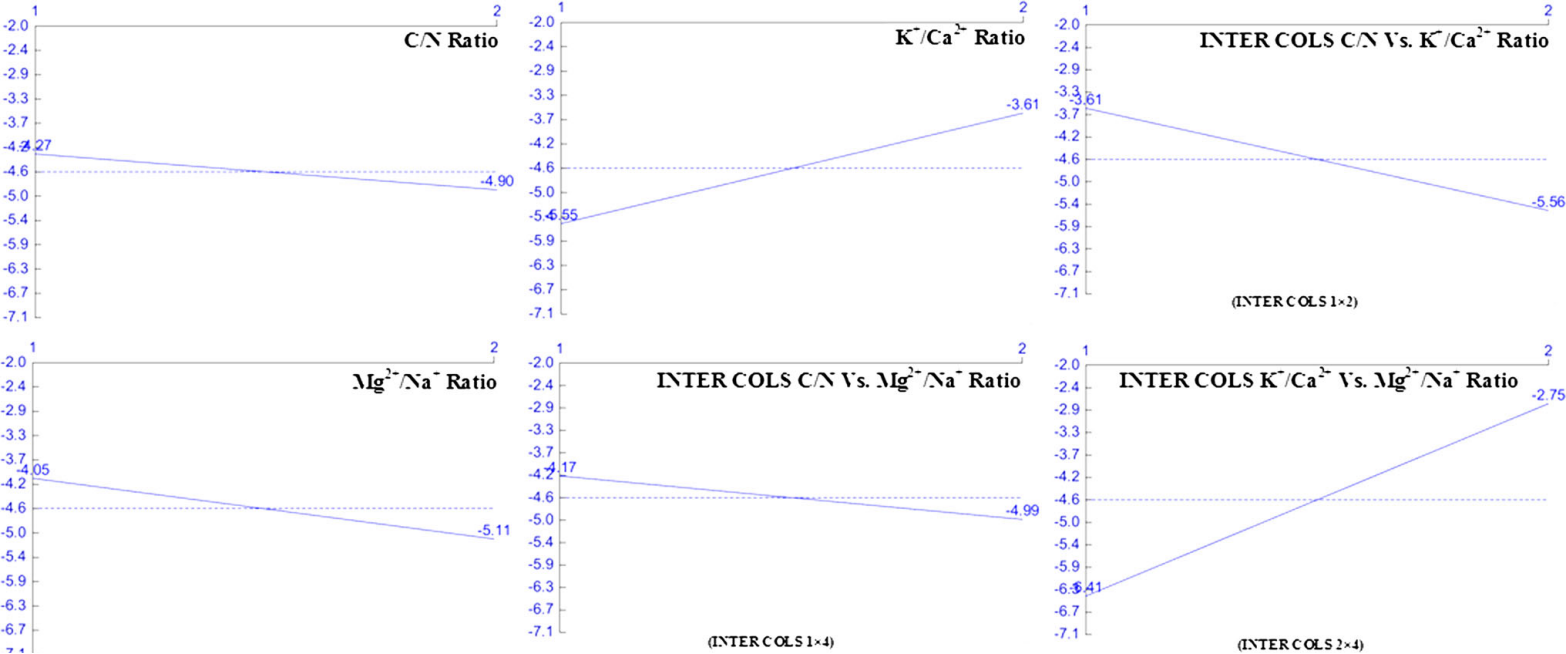
Impact of selected factor at their assigned ratio *Y*_p/x_ by *A. giganteus* MTCC: 8408 in submerged fermentation

The main effect of the fermentation factors at their assigned ratio on *Y*_p/x_ are described in Table 3. It shows the interaction with factor’s average effect at their individual assigned ratio on higher *Y*_p/x_ by *A. giganteus* MTCC: 8408 in submerged fermentation. It can be seen from the Table 3 that INTER COLS 2 × 4 (*L*_2_ − *L*_1_ = 3.662) showed stronger influence compared to other factors followed by K^+^/Ca^2+^ level (*L*_2_ − *L*_1_ = 1.943) in afp production. Figure 1 represents the influence of average effect of factors on afp production by *A. giganteus* MTCC: 8408 in submerged fermentation. At individual ratio, the higher *Y*_p/x_ was observed with INTER COLS 2 × 4 at level 2 (−2.751) followed by INTER COLS 1 × 2 at level 1 (−3.607).

**Table 3:** Average effects (main effects) of selected factors on *Y*_p/x_

Columns	Factors	Level 1	Level 2	*L*_2_ − *L*_1_
1	C/N ratio	−4.267	−4.897	−0.63
2	K^+^/Ca^2+^	−5.553	−3.61	1.943
3	INTER COLS 1 × 2	−3.607	−5.557	−1.951
4	Mg^2+^/Na^+^	−4.049	−5.115	−1.066
5	INTER COLS 1 × 4	−4.169	−4.995	−0.827
6	INTER COLS 2 × 4	−6.413	−2.751	3.662

Increase in C/N ratio ensued a mere reduction in *Y*_p/x_ up to level 2 (−4.267 to −4.897) while at individual level, increase in Mg^2+^/Na^+^ ratio ensued significant decrease in *Y*_p/x_ up to level 2 (−4.049 to −5.115). All other factors under this category showed variable effect on afp production, suggesting the selected factors and their levels were within the ideal average conditions. Presence of mono- and divalent cation had significant effect on cell morphology. The effect of K^+^/Ca^2+^ and Mg^2+^/Na^+^ ratio on *Y*_p/x_ was inversely proportionate. Increase in K^+^/Ca^2+^ ratio caused abrupt increase in *Y*_p/x_ up to level 2 (−5.553 to −3.61) while decrease in INTER COLS 2 × 4 had higher effect on *Y*_p/x_ at level 2. The impact of factors on higher *Y*_p/x_ was followed as: INTER COLS 2 × 4 (at level 2) > INTER COLS 1 × 2 (at level 1) > K^+^/Ca^2+^ (at level 2) > Mg^2+^/Na^+^ (at level 1) > INTER COLS 1 × 4 (at level 1) > C/N ratio (at level 1).

### % Severity index (SI)

**Fig. 2:**
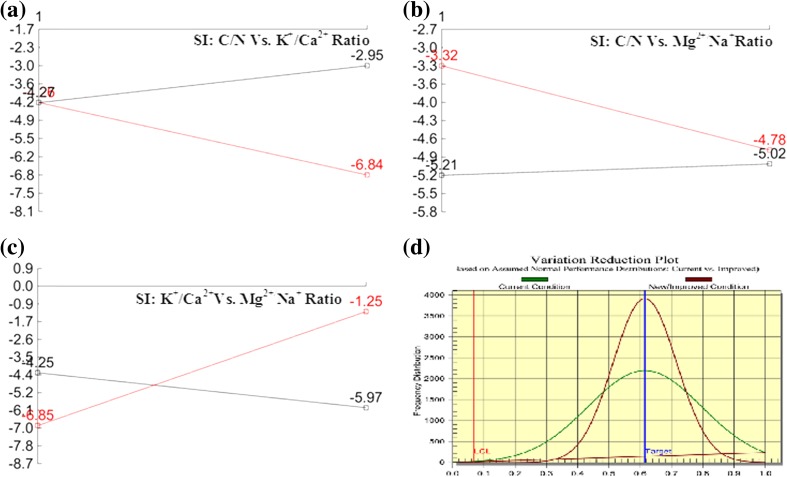
Severity index interaction plot and performance distribution plot

**Fig. 3:**
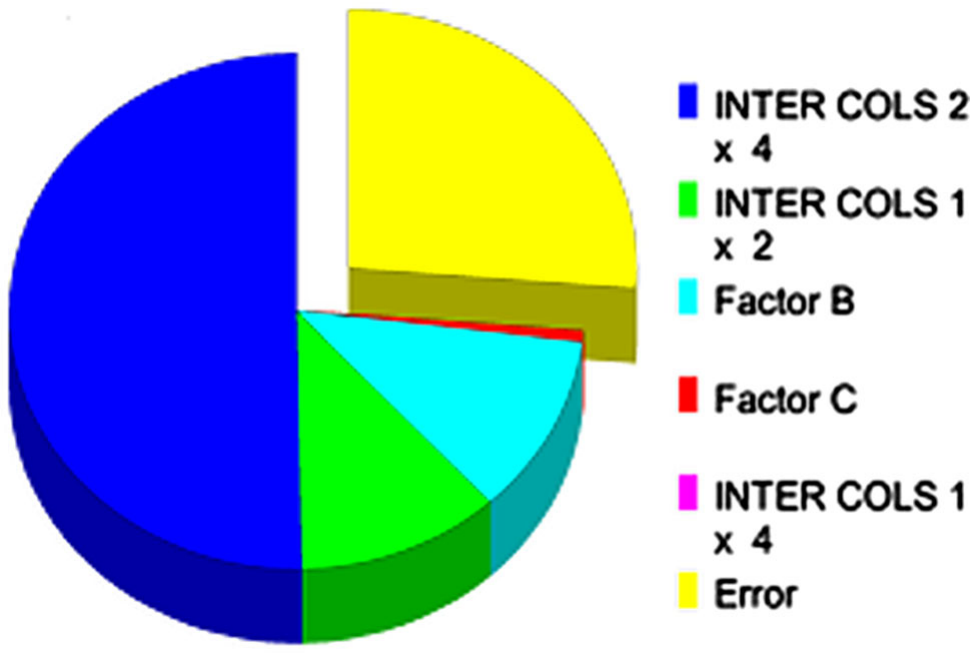
Relative influence of factors and interactions at their assigned ratio

To have a better insight on the overall DOE analysis and the possibility of presence of most interactions, severity index (SI) study was evaluated (Fig. 2) from Taguchi DOE L_8_ OA projection that represents the influence of two level factors with interaction (outer array) at their assigned ratio for higher *Y*_p/x_. The highest interaction; SI 65.32% (Fig. 2c) was appeared between K^+^/Ca^2+^ and Mg^2+^/Na^+^ (at level 1) (at levels 2 and 1; reserved column 6) among the all interactions followed by SI 50.08% (Fig. 2a) between C/N ratio and K^+^/Ca^2+^ (at level 2 and 2; reserved column 3) and SI 43.67% (Fig. 2b) between C/N ratio and Mg^2+^/Na^+^ (at level 1 and 1; reserved column 5). It was interesting to observe that K^+^/Ca^2+^ ratio (-3.61 at level 2) and Mg^2+^/Na^+^ (−4.049 at level 1) with high impact factor showed highest SI in combination. On the contrary, the SI interaction between low impact factors C/N ratio (−4.267 at level 1) and Mg^2+^/Na^+^ (−4.049 at level 1) was less than 50% (at levels 1 and 1; reserved column 13). It was apparent from the observations that the influence of individual factors on *Y*_p/x_ had varying effects while in combination; the afp production was entirely independent of the individual influence. Figure 3 shows the relative influence of factors and interactions on afp production at chosen levels. Soluble starch has been shown to exert maximum positive impact on the production of afp production in individual cases.

### Analysis of variance (ANOVA)

ANOVA study (Table 4) showed that among all selected factors INTER COLS 2 × 4 contributed maximally (47.96%) on the overall *Y*_p/x_ followed by Mg^2+^/Na^+^ ratio (12.945%) and INTER COLS 1 × 4 (10.151%). All the factors and interactions considered in the experimental design were statistically significant with 90% of confident limit. K^+^/Ca^2+^ and INTER COLS 1 × 2 showed almost equal contribution of 9.037 and 9.136%, respectively. Negligible contribution was observed with C/N ratio (5.183%) at the individual level on overall production of afp under the aforementioned fermentation conditions, even though it has major impact on mass transfer of nutrients during fungal growth. This study revealed that overall 60.905% contribution was noticed with only two selected parameters (INTER COLS 2 × 4 and Mg^2+^/Na^+^ ratio) and rest 33.507% by other selected factors (negligible error, 5.588%). From ANOVA evaluation, the impact of factors on the overall afp production was observed as: INTER COLS 2 × 4 > Mg^2+^/Na^+^ ratio > INTER COLS 1 × 4 > INTER COLS 1 × 2 > K^+^/Ca^2+^ > C/N at their assigned individual ratio under the Taguchi DOE L_8_ OA projection.

**Table 4:** Analysis of variance (ANOVA)

Columns	Factors	DOF	Sum of sqrs.	Variance	F-ratio	Pure sum	% contribution
1	C/N ratio	1	0.795	0.795	0.258	2.541	5.183
2	K^+^/Ca^2+^	1	7.553	7.553	2.451	4.471	9.037
3	INTER COLS 1 × 2	1	7.602	7.602	2.467	4.52	9.136
4	Mg^2+^/Na^+^	1	2.27	2.270	0.736	5.346	12.945
5	INTER COLS 1 × 4	1	1.365	1.365	0.443	3.976	10.151
6	INTER COLS 2 × 4	1	26.812	26.812	8.700	23.731	47.960
Other error		1	3.081	3.081			5.588
Total		7	49.481				100

### Optimized conditions and validation

The optimum conditions and their contribution are shown in Table 5. Based on detailed analysis, optimized condition for maximum afp production could be at C/N ratio: 27.4:1; K^+^/Ca^2+^ ratio: 4.78:1; Mg^2+^/Na^+^ ratio: 0.47:1; The expected *Y*_p/x_ at optimum conditions was 1.054 (based on SN = 0.457) with total contribution from all the factors was 1.78 (based on SN = 5.035) with grand average performance of 0.59 (based on SN = −4.582 mg L^−1^). The observed 68.88% grand average performance of the fungal strain and 44.02% contribution of all fermentation factors revealed the potential of the fermentation factors concentration and their interaction for afp production by the fungus, *A. giganteus* MTCC: 8408. Figure 2d depicts the performance distribution of current condition along with improved condition. The overall enhancement of *Y*_p/x_ was 44.02% i.e. from 0.59 to 1.054 can be achieved. Further to validate the proposed DOE, experiments were performed for afp production by employing the optimized culture conditions (Table 5). The experimental data showed an enhanced *Y*_p/x_ yield by 47.32% i.e. from 0.59 (based on SN = −4.582 mg L^−1^) to 1.12 with mycelial biomass 23.9 ± 3.9 gdcw L^−1^, thus proving the validity of the method with the new modified culture conditions.

**Table 5:** Optimum culture conditions and their contribution

Columns	Factors	Level description	Level	Contribution
1	C/N ratio	27.4:1	1	0.315
2	K^+^/Ca^2+^	4.78:1	2	0.971
3	INTER COLS 1 × 2	–	1	0.974
4	Mg^2+^/Na^+^	0.47:1	1	0.532
5	INTER COLS 1 × 4	–	1	0.413
6	INTER COLS 2 × 4	–	2	1.830
	Total contribution from all	Factors		5.035
	Current grand average	Performance		−4.582
	Expected result at optimum	Condition		0.457

### Fermentation factors on morphological differentiation and various biochemical yield

**Fig. 4:**
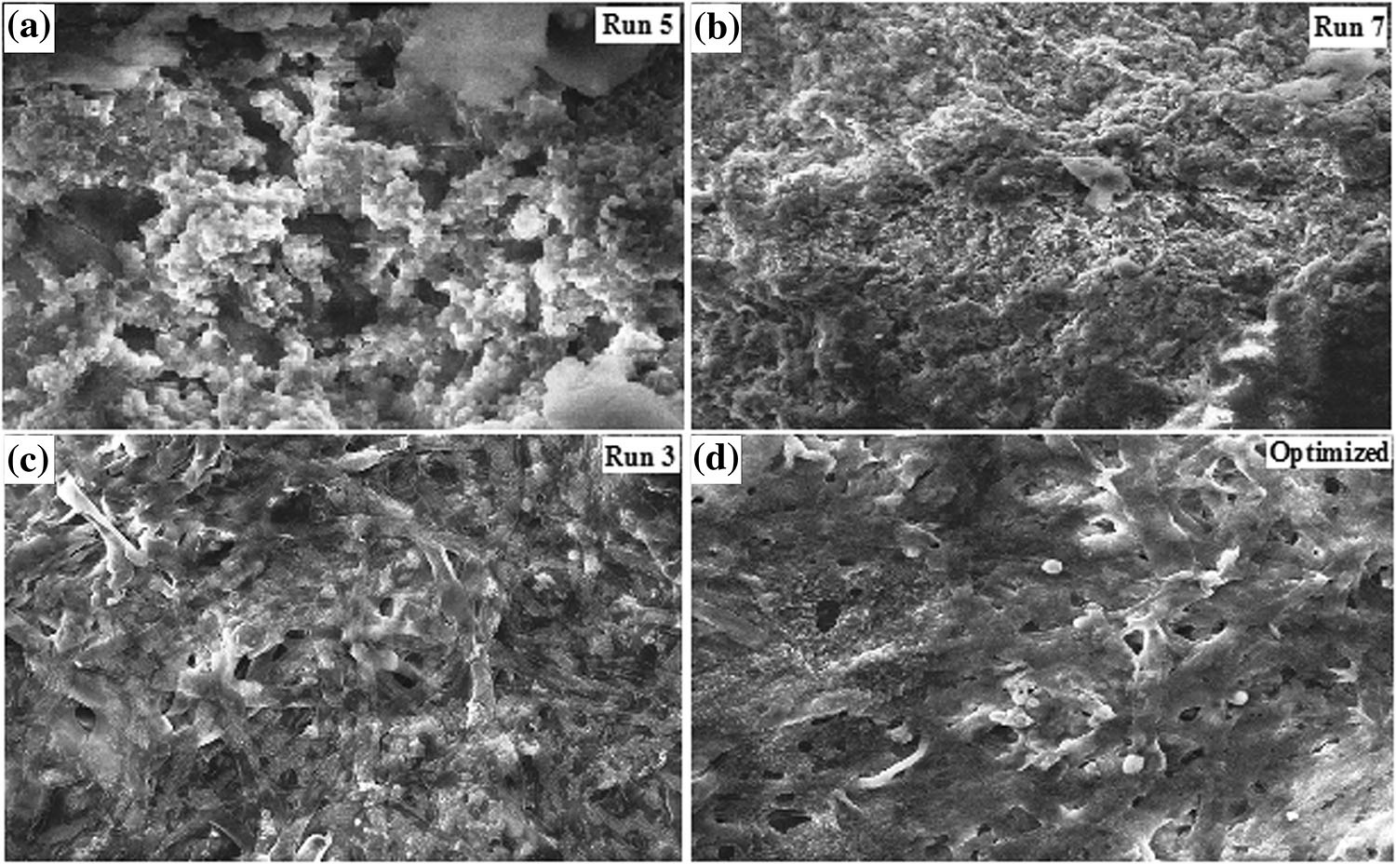
Morphological differentiation of *Aspergillus giganteus* MTCC 8408 cell on nutrient dynamics and optimization. Lateral resolution: 2–10 µm; magnification: 5KX; EHT: 18.00 kV; WD: 9.5 mm

The slowly utilizable soluble starch which favors the secondary metabolite production resulted lower level of *µ* value but with higher accumulation of intracellular afp in the cell due to the higher *Y*_p/x_ and µmax value (Table 6). Run-3 produced the highest *Y*_p/x_ and *Y*_p/s_ than run-5 in our study. This indicated that soluble starch with low C/N ratio (27.4:1) was propitious to the release of catabolite repression. Run-7 with high C/N ratio (54.9:1) resulted in second highest *Y*_p/x_ value with least *µ* value, indicating the effects of catabolite repression was decreased in afp biosynthesis. The influence of various C/N ratio including microelements on afp production was directly controlled and regulated by fungal morphological differentiation (Fig. 4). In slowly utilizable soluble starch and corn steep liquor with proteose peptone mycelium was developed thriftily to bigger and looser coarse form with thicker hairy outer region. Tardy assimilation of soluble starch not only supports growth but also self maintenance and survival, compact and bulbous, spongy filamentous with too much sporangium on the tips of hyphae. Highest value of *µ* was obtained at Run-1 (1.63 ± 0.35) with low C/N ratio (27.4:1) resulted in second highest *Y*_x/s_ and second lowest *Y*_p/s_ value. This indicated that the faster cell growth resulted in undesired fungal morphology which was inconsistent with the requirement of nutrients (Fig. 4a). Therefore, the carbon catabolite repression occurred in the process with high C/N ratio (54.9:1) in the media. The slowly utilizable soluble starch was easier to induce fungal morphological differentiation which was proved to be propitious to the secondary metabolism.

**Table 6:** Influence of C/N, K^+^/Ca^2+^ and ratio Mg^2+^/Na^+^ on biochemical parameters of various yield in submerged fermentation of *Aspergillus giganteus* MTCC 8408 as per Taguchi DOE L_8_ OA

Parameters	Run 1	Run 3	Run 5	Run 7	Run 8
Max. biomass (gdcw L^−1^)	25.05 ± 0.35	22.10 ± 0.78	29.85 ± 0.80	21.05 ± 0.42	26.15 ± 0.23
*Y*_x/s_ (gdcw g^−1^ L^−1^)	0.835	0.736	0.995	0.69	0.66
*µ* (D^−1^)	1.63 ± 0.35	1.55 ± 0.31	1.21 ± 0.12	1.11 ± 0.24	1.15 ± 0.23
*Y*_p/x_ (mg gdcw^−1^ L^−1^)	0.578	0.882	0.385	0.878	0.592
*Y*_p/s_ (mg g^−1^ L^−1^)	0.483	0.65	0.383	0.616	0.516

Na^+^ stimulates dry weight production at low concentrations (Run-5) while K^+^ inhibits it at high concentrations (Run-3) resulted filamentous form of growth was maintained with the hyphae being scarcely branched without bulbous cells (Fig. 4c). Magnesium, calcium inhibits dry weight production at all concentrations (Run-2 and Run-7). The inhibition of dry weight production by sodium was encountered by magnesium and calcium at higher K^+^/Ca^2+^ ratio and lower Mg^2+^/Na^+^ ratio (Run-7) resulted boisterous distribution, broken hyphal tips and concomitant cell wall immaturation (Fig. 4b). Minimum concentration of calcium ions reduced the extent of the inhibition of growth brought about by sodium ions was 1.08 m-equiv, while the increased growth in the presence of added calcium ions of 3.2432 m-equiv resulted pelleted type morphology with significant reduction of *Y*_p/x_ value (Fig. 4a). Magnesium was also as effective as calcium in reducing the extent of the inhibition brought about by sodium ions and the minimum concentration was 3.252 m-equiv. Addition of 1.08 m-equiv L^−1^ Ca^2+^ ions to the fermentation medium lowered the final dry cell weight by 22% as compared to 3.2432 m-equiv L^−1^ and increased the uptake of phosphate and sucrose, and afp production. At high K^+^/Ca^2+^ ratio (4.78:1) highly branched hyphae and numerous bulbous cells was observed (Fig. 4d) while laminated layers, featured vesicles associated with the numerous inclusions was observed at high Mg^2+^/Na^+^ ratio (0.94:1). Ca^2+^ competed with magnesium and prevented growth almost entirely when present at 4.51 times in excess.

## Discussion

The C/N ratio is important for any fermentation process. A proper C/N ratio for pure culture is necessary to optimize aerobic fermentation in submerged condition from organic substrate. It is therefore, necessary to maintain proper composition of the feedstock for efficient scale up (Adour et al. 2002).

Linear, soluble and amylopectin fractions made soluble starch excellent for growth and biochemical yield. CSL, a by-product of corn-processing industry, comprised of peptides, sugars, lactic acids, vitamins and metallic ions and improved growth during early stages of colonization (Li and Holdom 1995; Enoch et al. 2001). During fermentation, these are taken up and directly incorporated or transformed into other cellular metabolites of interest (Liu and Chen 2002). By contrast, proteose peptone induced cell spends more energy and less time during lag phase in synthesizing amino acids for protein from organic sources. At higher C/N ratio 54.9:1 maximum biomass obtained in this study was in Run-5 (29.85 ± 0.80 gdcw L^−1^) which was higher than in Run-7 and Run-8 while *Y*_p/x_ yield reached maximum in Run-3 (0.882 mg gdcw^−1^ L^−1^) but comparable to Run-7 (0.878 mg gdcw^−1^ L^−1^). We attributed this decreased in biomass was due to high K^+^/Ca^2+^ ratio in Run-7 as compared in Run-5 that lead to enhancement *Y*_p/x_ but reduced *µ* value. In this study, we found that at optimum C/N ratio of 27.4:1, afp yield was the maximum (*Y*_p/x_ = 1.12 mg gdcw^−1^ L^−1^) and reached to 25.98 mg L^−1^. This study demonstrated that at high Mg^2+^/Na^+^ ratio (0.94:1) growth may be prevented and may be competed with Ca^2+^ almost entirely when present at 4.51 times in excess.

## Conclusion

The influences of various C/N ratios with four essential microelements on fungal morphological differentiation and afp production were investigated in submerged fermentation of *Aspergillus giganteus* MTCC 8408. The amylopectin fractions in soluble starch with corn steep liquor and proteose peptone comprised of peptides, sugars, lactic acids, vitamins and metallic ions affected afp production by both regulating biosynthesis and inducing fungal morphological differentiation. Although, the physiological mechanism of action of the K^+^ ion in cell growth is not understood, ratio of K^+^/Ca^2+^ played a major role in endogenous cell differentiation and enhanced afp production. Another interesting point can be seen in Table 6 that the effect of lower levels of magnesium is greater on µ (1.63 ± 0.35) in Run-1 than µ (1.15 ± 0.23) in Run-8. This would mean moderate shortages of magnesium would tend to affect the onset of fermentation rather than the eventual degree of attenuation. Published data suggest that effects of C/N ratio with these alkali and alkaline earth metal ions can have great significance in commercial practice.
